# Ibn al-Haytham's ground theory of distance perception

**DOI:** 10.1177/20416695221118388

**Published:** 2022-09-04

**Authors:** H. A. Sedgwick

**Affiliations:** State University of New York, State College of Optometry New York, New York, NY, USA

**Keywords:** 3D perception, distance perception, al-Haytham, Alhazen, J. J. Gibson, extended surfaces, depth perception, scene perception

## Abstract

The 11th-century Arab scholar, Ibn al-Haytham, in his *Optics*, offers a detailed, rigorous, empirically oriented explanation of distance perception that may be the first essentially modern, scientific theory of distance perception. Based on carefully described experiments, he argues that for distance to be perceived accurately: (1) the distance must lie along a continuous surface such as the ground; (2) the continuous surface must be visible; (3) the magnitudes of distances along the surface must be perceived and calibrated through bodily interaction (walking and reaching) with them; and finally (4) the distance must be moderate. Al-Haytham's work reached Europe early in the 13th century, and his was the dominant theory of distance perception there for about 400 years. It was superseded early in the 17th century by a theory, based on cues such as convergence and accommodation, of distance seen through empty, mathematized space. Around 1950, an explanation of distance perception strikingly like that of al-Haytham was independently developed by J. J. Gibson, who called his theory the “ground theory” of space perception.

## Introduction^1^

Ibn al-Haytham, who was called Alhazen in medieval Europe, was an Arab scientist and philosopher. He was born around 965 C.E. in the city of Basra in what is now Iraq, but he moved to Cairo where he settled near the Al-Azhar Mosque and spent the remainder of his life studying, writing, and teaching. Al-Haytham wrote around 90 works—of which over 60 are still extant—on theology, physics and metaphysics, mathematics, optics, astronomy, natural philosophy, and medicine. He died around 1039 C.E. (al-Haytham, 1989).

This paper discusses what I am calling Ibn al-Haytham's “ground theory” of distance perception.^2^ The term “ground theory” is not al-Haytham's. It comes from James J. Gibson, who proposed what he called the “ground theory of space perception” in 1950 (Sedgwick, 2021). In what he saw as a “radical reformulation” of existing theories, Gibson hypothesized that

[T]here is literally no such thing as a perception of space without the perception of a continuous background surface. (p.6)^3^

Doing research during World War II on the distance perception of pilots while landing airplanes, Gibson became dissatisfied with the prevailing approach to studying space perception. As he described it:

The theories of space-perception which flourished in the 19th century were all theories of abstract, empty space. (p. 60)

The classical theories of space perception conceived the third dimension to be a line extending outward from the eye. Space was therefore empty between the eye and the object fixated. (p. 61)

**Figure 1.**
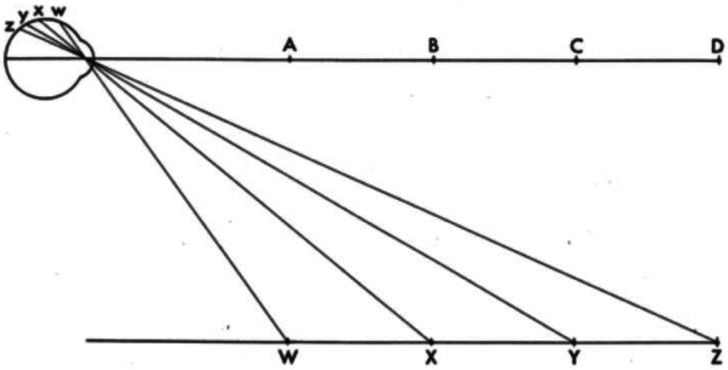
“Two formulations of the problem of distance perception.” (Gibson's caption. Adapted from Gibson, 1950).

Gibson contrasted his new theory and the classical theories diagrammatically, as shown in Figure 1. The upper horizontal line illustrates the classical theory.^4^ In the projection of this line onto the retina, as Gibson observes,

The points A, B, C, and D are not discriminable on the retina. (p. 61)

For the classical theory, distance perception is a problem because distance is thought of as a line projecting end-on to the eye (Berkeley, 1910/1709). The third dimension—the dimension of distance—is lost in the retinal projection.

In Gibson's theory, by comparison, the optical projections of distances along with the ground are spread out on the retina; nothing is lost. Thus, distance perception does not pose a special problem for visual perception.

The basic idea is that visual space should be conceived not as an object or an array of objects in air but as a continuous surface or an array of adjoining surfaces. The spatial character of the visual world is given not by the objects in it but by the background of the objects. It is exemplified by the fact that the airplane pilot's space, paradoxical as it may seem, is determined by the ground and the horizon, not by the air through which he flies. (p. 6)

The lower line in Figure 1 illustrates Gibson's ground theory. As he notes,

The points W, X, Y, and Z at corresponding distances *are* discriminable on the retina. They represent the image of an extended surface… (p. 61)

Although Gibson's ground theory was, as he said, “new at least to the writer” (p.6), it was not new. A strikingly similar account had been laid out in detail by Ibn al-Haytham, over 900 years earlier. In this paper, I first examine al-Haytham's formulation of the ground theory. Then I attempt to place al-Haytham's theory historically, looking for possible antecedents and asking what became of the ground theory after al-Haytham. What influence did it have? When and why was it lost from sight?

## Ibn al-Haytham's Ground Theory

Al-Haytham was in the public eye a couple of decades ago when Richard Powers, writing in The New York Times Magazine, credited him with the “Best Idea of the Millenium” for inventing the experimental method (Powers, 1999). Al-Haytham's use of this method is very much in evidence in his extensive work on *Optics*. The sections of this work dealing with light and with the structure of the eye have long been widely known and have been highly influential, but the *Optics* also deals extensively with visual perception; it is only with its translation from Arabic into English in 1989 (al-Haytham, 1989) that this aspect of his work has begun to be explored by contemporary vision scientists (Gilchrist, 1996; Howard, 1996; Wade, 1996). My discussion of al-Haytham's theory of distance perception is based on this English translation and is extracted from al-Haytham's detailed consideration of the perception of size, distance, and other geometrical properties of the three-dimensional world.

Here is al-Haytham's formulation of how we perceive distance. The words are different from Gibson's, but the meaning is very similar:

sight does not perceive the magnitudes of distances of visible objects from itself unless these distances extend along a series of continuous bodies, and unless sight perceives those bodies and their magnitudes. (p. 155)^5^

Al-Haytham describes an experiment to demonstrate this point. The following long quote (divided into 4 parts) conveys some of the care and rigor of al-Haytham's thinking (the words are al-Haytham's; the drawings are mine).

**Figure 2.**
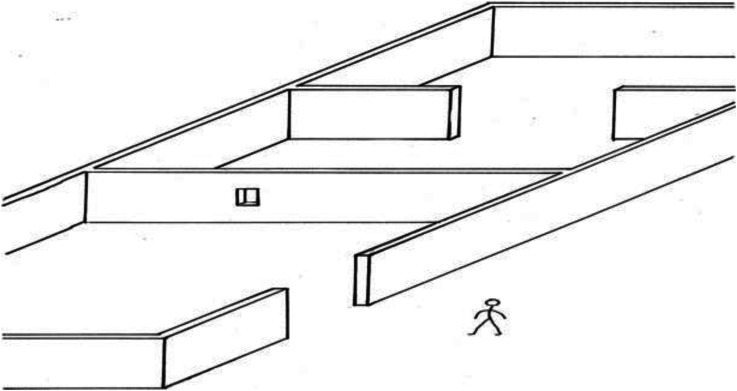
al-Haytham's aperture experiment [1].

Al-Haytham begins by describing the general setup (Figure 2):

[1] Let the experimenter go to a chamber or place which he has not entered before, and let there be a narrow hole^6^ in one of the walls of this chamber or place, and behind that hole let there be an open space which the experimenter has not previously observed. (p. 153)

**Figure 3.**
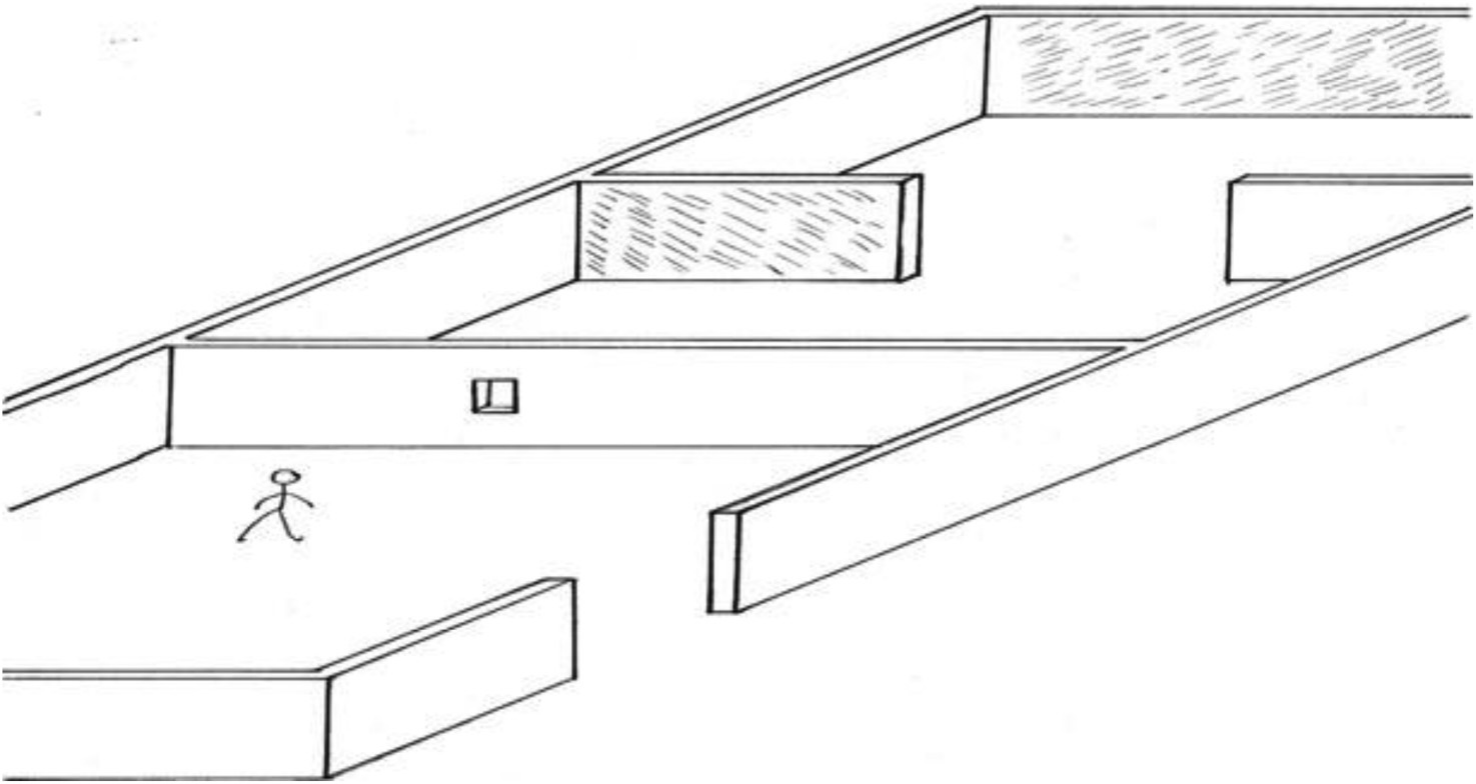
al-Haytham's aperture experiment [2].

Next, al-Haytham specifies the two walls whose distances from the observer are to be compared (Figure 3):

[2] Let two walls stand in that space so that one of them will be closer to the hole than the other. Let there be a sizable distance between the two walls and let the nearer wall hide part of the farther and let the other part of the farther wall be visible. (p. 153)

**Figure 4.**
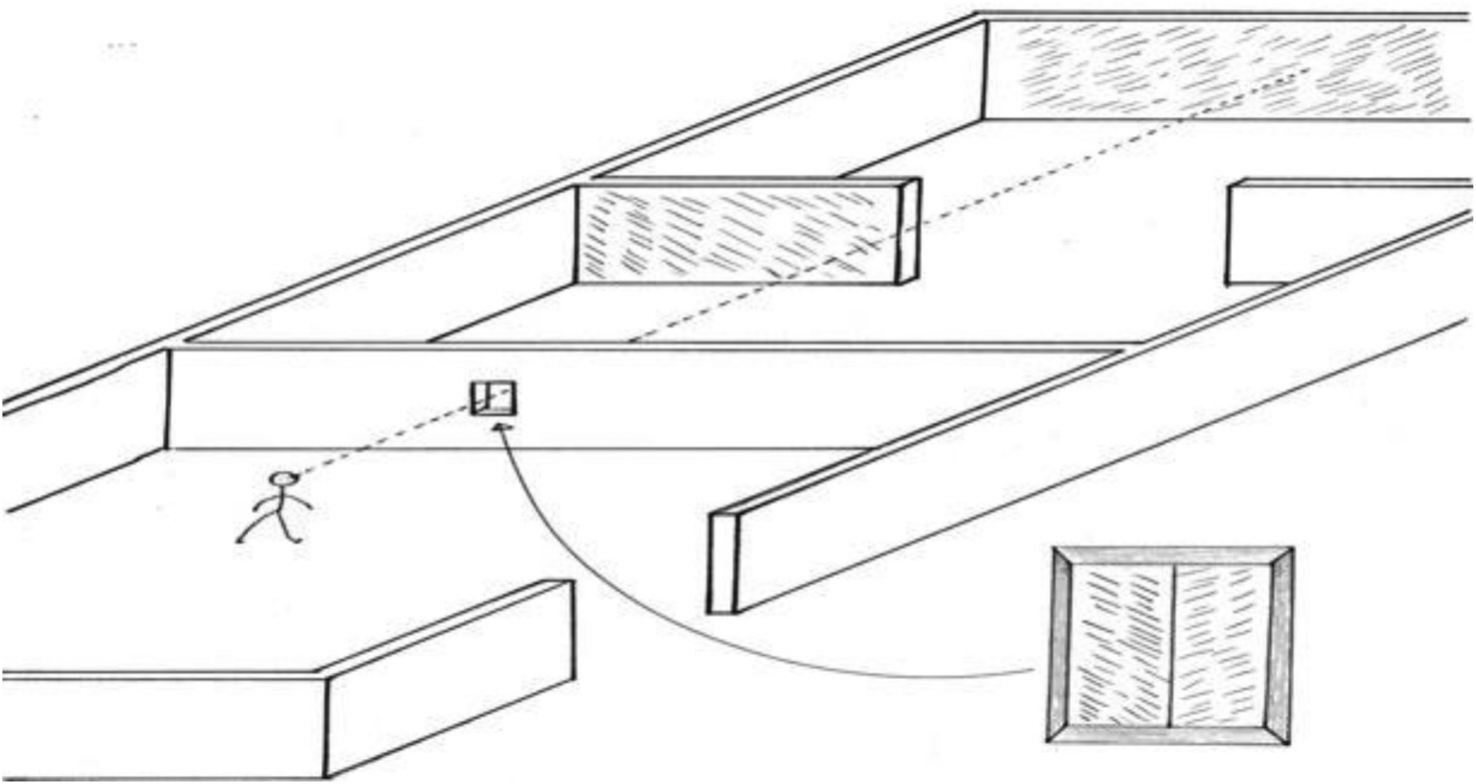
al-Haytham's aperture experiment [3] (inset shows observer's view).

Then, al-Haytham specifies the viewing condition and what is perceived (Figure 4):

[3] Let the hole be above the ground so that upon looking through it the observer will not see the ground-surface behind the wall that has the aperture. Having come to this place and looked through the aperture, the experimenter will see the two walls together without perceiving the distance between them. (p. 153)

Finally, al-Haytham describes the results of several variants of the experiment:

[4] If the distance of the first wall from the aperture is excessively large, he will perceive the two walls as contiguous and may take them to be a single, continuous wall if their color is the same. If the first wall is moderately distant from the aperture and the observer senses the two walls as two, he will take them to be close together or contiguous and fail to ascertain the distance between them. He will also perceive the first wall, if moderately distant, as if it were close to the aperture without ascertaining its distance either. (p. 153)

In al-Haytham's experiment, the observer fails to see the separation between the two walls because the ground between them is not visible. In describing his experiment, al-Haytham stipulates that the layout be unfamiliar, but he then briefly introduces the possibility of what contemporary theory calls “informational encapsulation” (Fodor, 1983) or, more specifically, “cognitive impenetrability” (Pylyshyn, 1999):

sight may perceive two such bodies as being contiguous, even if it had previous knowledge of the distance between them. (p. 154).

**Figure 5.**
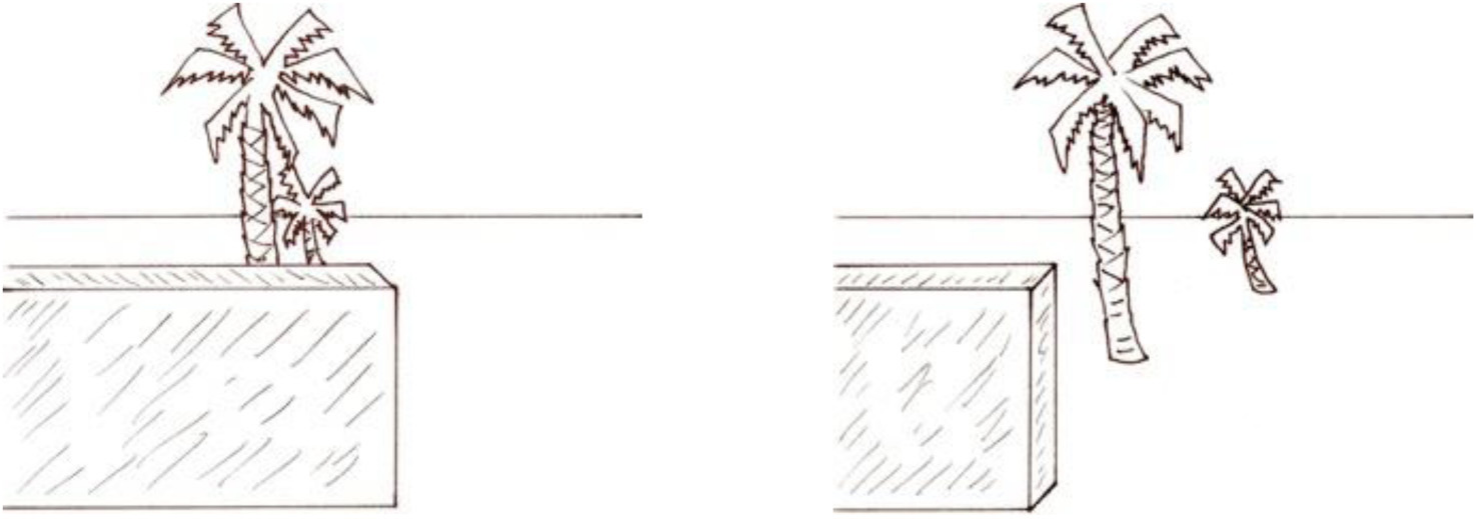
Distance perception without or with a visible ground.

In another, more briefly described experiment, al-Haytham contrasts situations in which the ground is, or is not, visible (Figure 5):

When an observer looks at two persons (or poles or palm trees) standing on the ground, with a sizable distance between them, and one of them appears to hide part of the other, but the observer does not perceive the ground between them, and assuming that he has not previously seen those two poles or persons, and that the far person is not excessively distant, then upon looking at them together he will take them to be contiguous, or with a small distance between them, and will not sense the magnitude of their distance from each other. When he then changes position so as to see the continuous ground between them he will perceive the distance of the far person [from himself] and the distance between the two persons, and become aware of the sight's error in the first perception. (p. 154)

**Figure 6.**
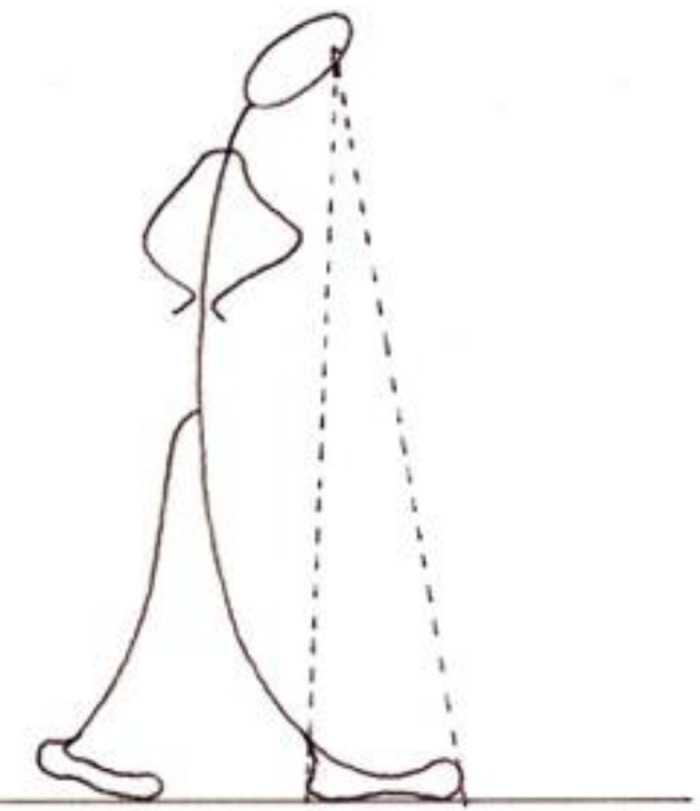
Perceiving the scale of the ground by comparison with the foot.

Having shown that a visible continuous surface such as the ground allows the accurate perception of distance, it remains for al-Haytham to explain how it does so. According to al-Haytham, the perception of distance is learned in early childhood and begins with those parts of the ground that are closest to the observer. The magnitudes of those parts are perceived relative to the body of the observer and become familiar with repeated experience (Figure 6):

For we always measure such parts unintentionally by our feet whenever we step upon them, or by our arms whenever we stretch our hands to them. Thus all parts of the ground next to us are always measured unintentionally by our body. (p. 180)

**Figure 7.**
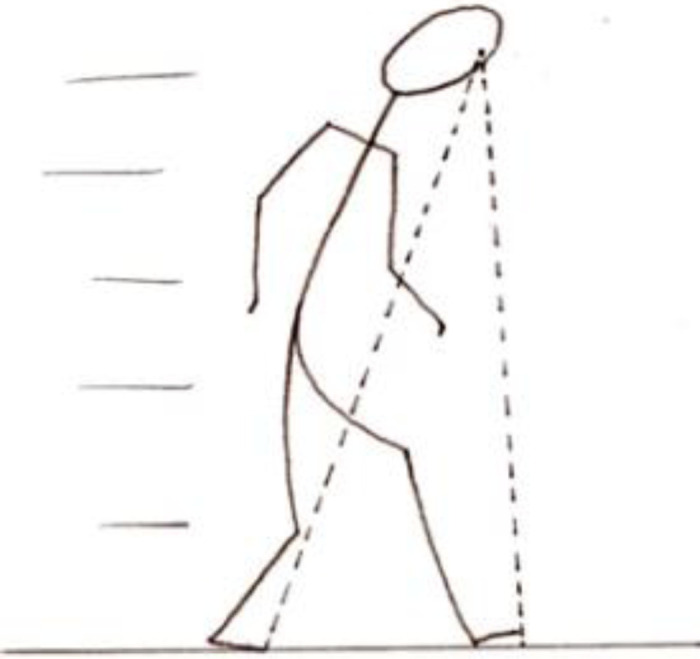
Extending the scaled portion of the ground by our steps when walking.

As we move around we extend our experience to surrounding parts of the ground, measuring the ground with our stride^7^ (Figure 7):

Again, the parts nearest those surrounding our feet are also measured by our body. For when we walk we measure the part of the ground on which we walk by our feet and our steps, and the faculty of judgement perceives that part's magnitude. (p. 181)

**Figure 8.**
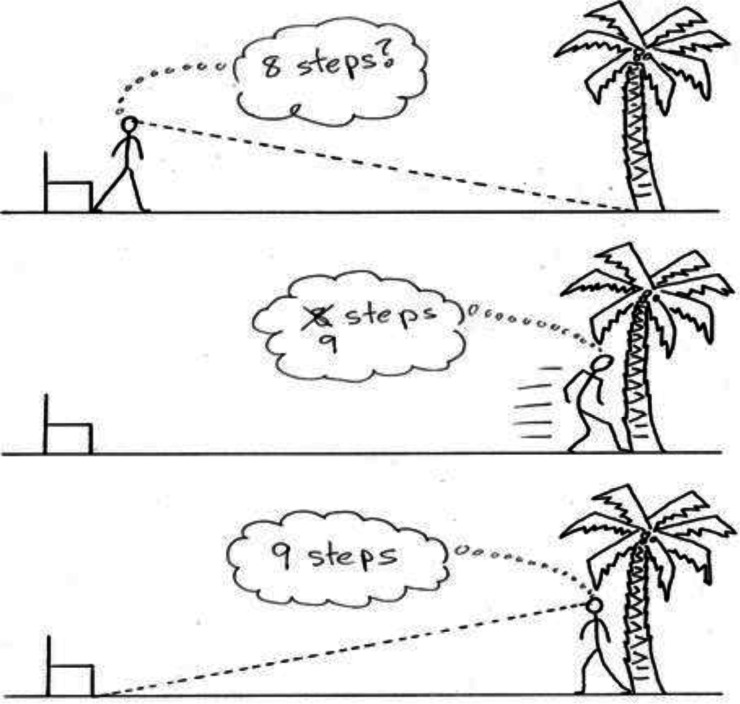
Calibrating the accurate perception of distance through experience.

Al-Haytham goes on to describe a cycle of estimation, followed by verification, followed by repeated estimation. Seeing a part of the ground in the distance, we estimate its magnitude. Then, by walking up to it we check our estimate and correct it if necessary. With repeated experience, we eliminate the errors in our initial estimates. The correct perception of distance thus begins with the ground nearest to us and gradually, with increasing experience, extends to more and more remote parts of the ground. Alhazen is describing a process of calibration through which perceived extents on the ground become accurately scaled to our own bodies (Figure 8).

The accurate perception of distance is limited, however, according to al-Haytham, to moderate distances:

For sight does not distinctly perceive visible objects whose distances are immoderate. And if ordered and continuous bodies exist between the eye and these objects, sight will not distinctly perceive all of these bodies, and consequently will not distinctly perceive the intervals between their extremities, and therefore will not distinctly perceive the distances that separate [the eye] from the visible objects at the extremities of those bodies. (p. 152)

To summarize, four related conditions must be met if distance is to be perceived accurately. (1) The distance must lie along with a continuous surface; (2) the continuous surface must be visible; (3) the magnitudes of the continuous series of parts or bodies making up the surface must be perceived and calibrated through bodily interaction (walking and reaching) with them; and finally (4) the distance must be moderate.

“If the distances of visible objects [meet all these conditions]”, according to al-Haytham, then “sight will perceive their magnitudes correctly and with certainty”. He is careful here to explain what he means by “certainty”:

By “certainty” I mean the utmost of what the sense can perceive (p. 152).

**Figure 9.**
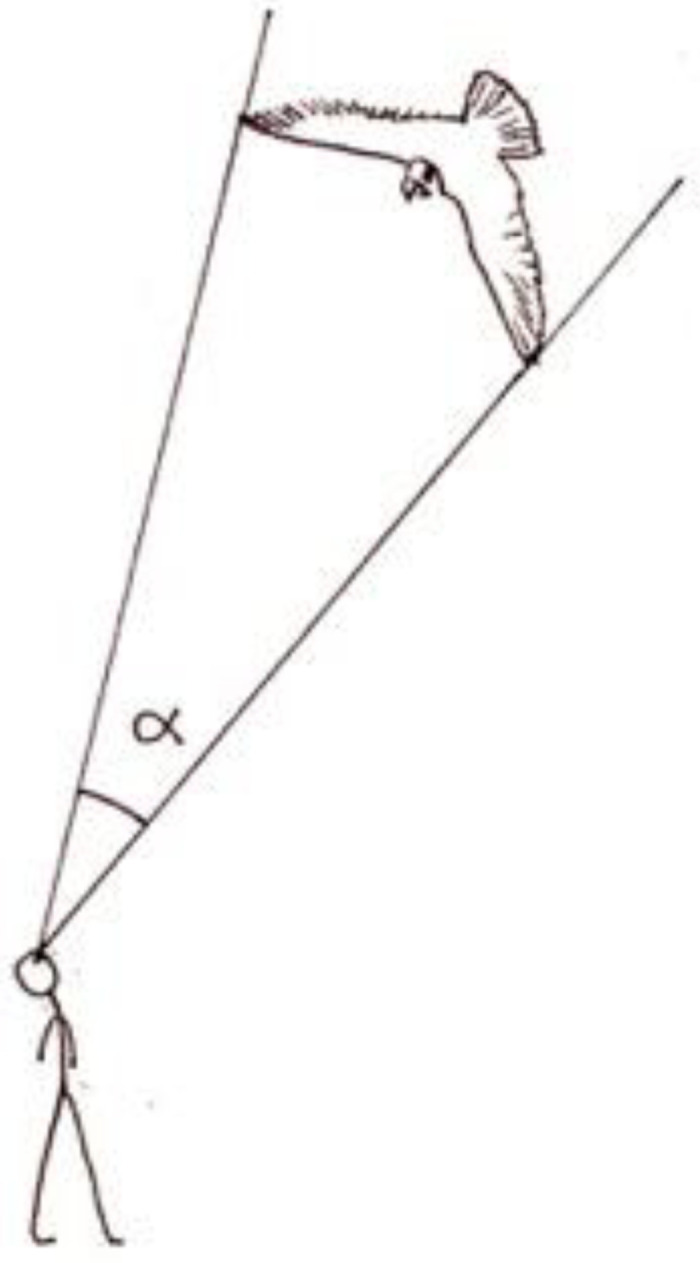
Conjecturing distance based on visual angle and familiar size.

When all of the conditions are not met for perceiving the magnitude of distance with certainty, then the faculty of judgment *conjectures* the magnitude of distance. Generally speaking, it seems that to al-Haytham this process of conjecture may make use of whatever signs or cues to distance are available. If the object is familiar, for example, then its familiar size may be combined with its visual angle to form a conjecture of its distance. Or if the object is not familiar, its distance may be conjectured on the basis of objects of similar form whose distances have previously been ascertained. Such conjectures of distance are prone to error, but the observer is aware of whether the magnitude of distance is being perceived with certainty or is only being conjectured (Figure 9).

This brief account cannot do justice to the richness and rigor of al-Haytham's theory of distance perception but is enough to establish its outlines and its striking similarity to Gibson's ground theory of space perception. I want to turn now to the historical setting of al-Haytham's work.

## Ibn al-Haytham's Antecedents

Did the ground theory originate with al-Haytham? Briefly, the answer is yes, as far as I know. The list of possible sources that I have been able to examine is so incomplete, however, that this conclusion must be quite tentative.

To understand this early work, we need to remember that it was not until the 17th century that Kepler worked out the optics of the eye, in which the light from an object passes through a small opening at the front of the eye and is focused by a lens to form an upside-down image at the back of the eye (Kepler, 1604/2000). Prior to Kepler, the basic functioning of the eye was more or less mysterious, so much so that it was not known whether vision occurred by means of something traveling from the object to the eye (intromission) or by something reaching out from the eye toward the object (emission).

The Islamic study of optics was spurred by translations into Arabic from Classical Greek and Latin texts. Of those, the principle works on optics that have come down to us are those of Euclid and Ptolemy. Euclid's work, from around 300 B.C.E., is a detailed geometrical study of how objects and surfaces shape the patterns of visual rays. Euclid believed in emission, as is clear in Lindberg's translation of Euclid's first assumption (or axiom):

Let it be assumed that the rectilinear rays proceeding from the eye diverge indefinitely;… (Lindberg, 1976, p. 12)

It is also clear, at least to this reader, that when Euclid uses the word (in translation) “appear” he is referring to perceived visual angles and directions in what we now call the visual field.^8^ Here are two more of his assumptions (or axioms):

[T]hat those things seen within a larger angle appear larger, and those seen within a smaller angle appear smaller, and those within equal angles appear to be of the same size;

and that things seen within the higher visual range appear higher, while those within the lower range appear lower;… (Burton, 1945, p. 357)

**Figure 10.**
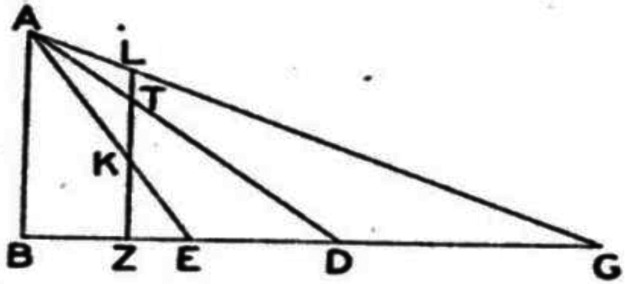
Diagram illustrating Euclid's proposition on height in the visual field. (Adapted from Burton, 1945).

The problem of distance perception is not mentioned by Euclid. One of his propositions, however, comes tantalizingly close to the ground theory. There is a striking similarity between Euclid's figure illustrating this proposition (see Figure 10) and Gibson's figure illustrating his ground theory (see the lower portion of Figure 1). Based on his assumptions, Euclid proves that:

In the case of flat surfaces lying below the level of the eye, the more remote parts appear higher. (Burton, 1945, p. 359)

By “higher,” Euclid clearly means higher in the visual field. From this proposition of Euclid's, one could easily derive a forerunner of the ground theory: the proposition that, for objects lying on the ground surface, those higher in the visual field are seen as farther away; but Euclid does not make such a move. Believing in the emission theory, Euclid may not have regarded distance perception as a problem.

Ptolemy, writing almost 500 years later, clearly asserted that we see by means of rays emitted from the eyes (Howard & Wade, 1996; Ross & Ross, 1976). For Ptolemy, this emission explains distance perception, we can directly sense the length of the ray by which we reach out to the object:

…longitudinal distance [is determined] by how far the rays extend outward…(Smith, 1996, p. 81)

whatever is seen with a longer ray appears farther away, as long as the increase in [the ray's] length is sensible [that is, can be sensed]. (Smith, 1996, p. 82)

Ptolemy's account of distance perception by emission could not have been any help to al-Haytham, who correctly believed that vision is produced by intromission: that is, rays (of light) coming from the object to the eye. As far as I have found, however, those ancient philosophers who believed in intromission were of no help either. We might look, for example, at the vivid account of distance perception given by Lucretius in his long poem *De Rerum Natura*. Following Democritus and Epicurus, Lucretius was an atomist. He believed that all objects are made up of tiny particles, called atoms and that they are continually shedding those atoms in all directions. As Epicurus (c. 270 B.C.E.) wrote:

For the flow of atoms from the surface of bodies is continuous, yet it cannot be detected by any lessening in the size of the object because of the constant filling up of what is lost. The flow of images preserves for a long time the position and order of the atoms in the solid body, though it is occasionally confused. (Oates, 1940, p. 6)

This is a theory of vision by intromission. These thin coats of atoms, like snake skins, physically convey an image of the object to the eye. Here is the intromissionist account that Lucretius (c. 55 B.C.E.) gives of distance perception:

And the image gives the power to see and the means to distinguish how far each thing is distant from us; for as soon as ever it is discharged, it pushes before it and impels all the air which lies between it and the eyes; and thus that air all streams through our eyes and brushes so to say the pupils and so passes through. The consequence is that we see how far distant each thing is. And the greater the quantity of air which is driven on before it and the larger the current which brushes our eyes, the more distant each different thing is seen to be. (Oates, 1940, p. 141)

The intellectual distance between this account of distance perception and al-Haytham's is impressive.

Islamic optics flourished for about 200 years before al-Haytham. My knowledge of Arabic texts from this period is limited to secondary sources, primarily a fairly detailed account by Lindberg (1976), which gives no hint of the ground theory before al-Haytham. According to him, works on optics by al-Kindi, Hunain ibn Ishaq, & al-Farabi mostly said that the visual rays come from the eyes (emission), from which I would tentatively assume that they did not regard distance perception as a problem. Al-Haytham himself gives no indication that the ground theory did not originate with him. To this, I would add that the care, the thoroughness, and the vigor of al-Haytham's exposition of the ground theory convey the strong impression that if he did not originate it, he certainly made it his own.

## al-Haytham's Followers

There is good evidence that for roughly 400 years the predominant theory of distance perception in Europe was al-Haytham's. Al-Haytham's *Optics* was translated into Latin, the scholarly language of Medieval Europe, early in the 13th century, and remained one of the most authoritative works on optics until the 17th century (Lindberg, 1967). Three of the principal indigenous European works on optics, those of Roger Bacon, Witelo, and John Pecham, all recite al-Haytham's description of distance perception. Bacon, c. 1267, wrote

…distance can be grasped and certified if it is moderate through the continuity of sensible bodies intervening between the eye and the distant object. (Lindberg, 1996, p. 207)

Bacon offers an example:

Thus when somebody is next to one wall and looks at another wall, beyond the first, higher than it, and at some distance from it, he does not perceive the distance between them, either because there is no continuous sequence of bodies between them or because he cannot perceive such bodies owing to the interference of the first wall, beneath which he stands. (Lindberg, 1996, p. 207, 209).^9^

Witelo, c. 1273, writes

No magnitude of distance therefore, of all visible things, is comprehended by the sense of sight alone, even with the help of the discerning faculty, except the magnitude of distance of those visible things whose distance extends along ordered and continuous bodies and whose distance is moderate. (Witelo, 1572/1972, p. 121)^10^

Finally, John Pecham, c. 1279, writes

Only moderate distances are certifiable to sight, and those by means of continuous and ordered intervening bodies. (Lindberg, 1970, p. 141).

Although Witelo's account is more extensive than the others, none of these European authors gives the impression of having been particularly interested in distance perception or engaged by al-Haytham's ground theory. Their recitations of it are brief and more or less unassimilated. In a mystifyingly syncretic move, for example, Pecham presents al-Haytham's intromission account of distance perception side-by-side with Ptolomy's emission account. These Medieval European authors add only a few, relatively minor contributions of their own, as far as I have found.

**Figure 11.**
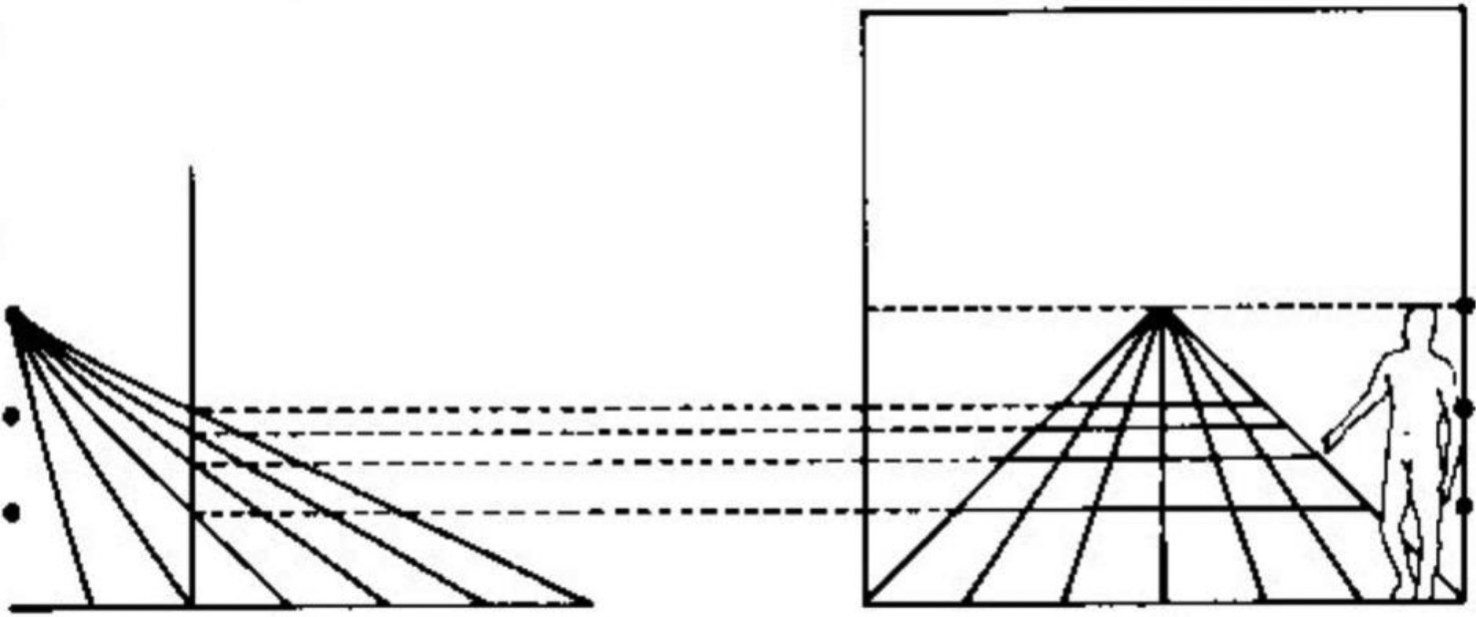
Alberti's diagram on how to construct a ground plane in perspective. (From Alberti, 1436/1972).

Beyond al-Haytham's influence on Medieval optics, there is the tantalizing possibility, raised by John White, that the development of pictorial space in the painting of Renaissance Italy, was directly influenced by al-Haytham's ground theory. The evidence for this is circumstantial, but it seems to me that a strong case can be made. Prior to the 14th century, pictorial space in European painting was flattened and highly stylized. Then, beginning in 14th century Italy, there was evidently an increasing interest in depicting greater depth and more realistic spatial layouts. Alberti's treatise *On Painting* (Alberti, 1436/1972), which is the first Renaissance account of how to create an accurate depiction of three-dimensional space, is highly congruent with al-Haytham's ground theory (see Figure 11).

**Figure 12.**
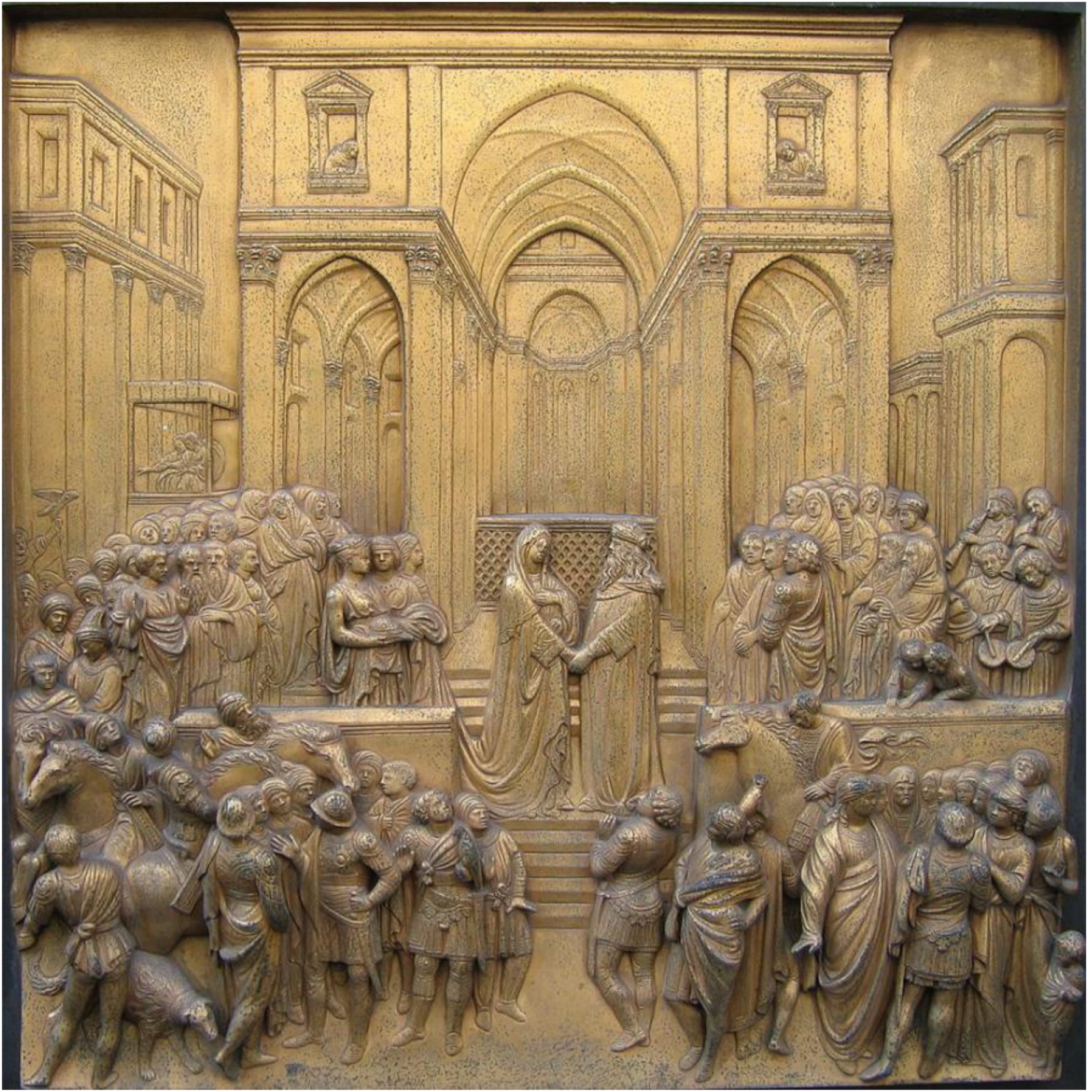
Solomon meets the Queen of Sheba, a panel from east doors of the Florence Baptistery by Lorenzo Ghibert, c. 1451. (Photo by Richardfabi from Wikimedia Commons.).

Using the human figure to scale his construction, Alberti describes a technique for constructing a regular perspective grid over the ground plane. This pavement provides the framework on which the scene is erected. Alberti makes little explicit reference to optics but suggests that he is familiar with it, saying that he is sparing the reader the geometrical demonstrations of the validity of his techniques. With Ghiberti, Alberti's contemporary in Florence, and the creator of the famous sculpted perspective panels on the bronze doors of the Baptistery, the connection is more directly demonstrable (Figure 12). Ghiberti's *Commentaries* include a series of propositions, excerpted from various optical texts, concerning the perception of size and distance; these include the following:

It is only possible to judge the distance of an object by means of an intervening, continuous series of regular bodies.

Distance is most commonly measured by the surface of the ground and the size of the human body.

(White, p. 127, quoting Ghiberti's excerpts from al-Haytham and Pecham)

Ghiberti died before finishing his *Commentaries*, and without making any explicit connection between these theoretical propositions and the newly developing practices of depicting spatial layout in painting and sculpture. Whether he might have done so had he lived to complete his *Commentaries* remains an open question.

## What Happened to al-Haytham's Ground Theory?

**Figure 13.**
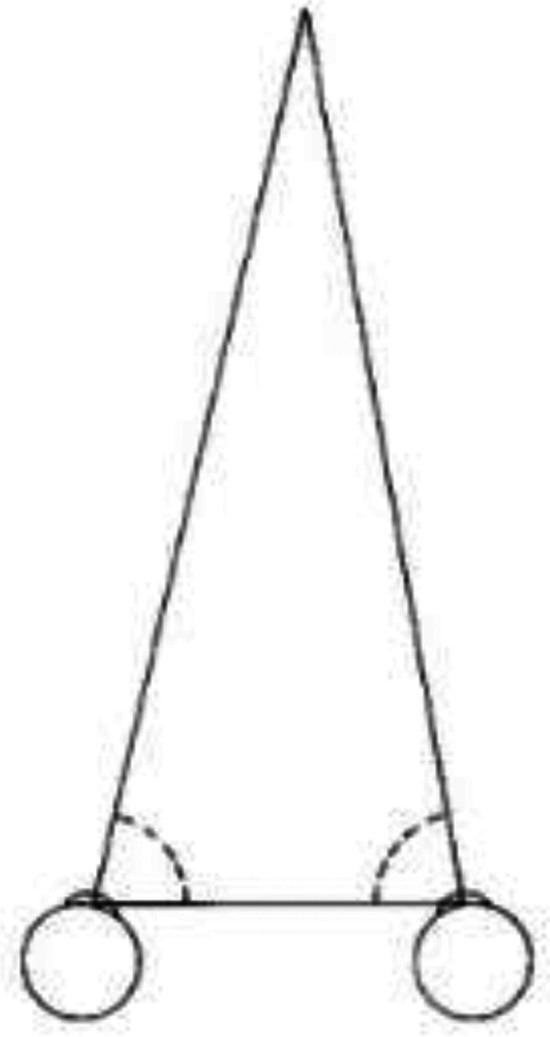
Distance derived from the geometry of ocular convergence.

The final question that I want to consider is what became of al-Haytham's ground theory? As we have seen, it was the dominant theory of distance perception in Europe until the end of the 16th century. As far as I have been able to determine it was never explicitly criticized or rejected. Instead, it was quietly abandoned following Kepler's introduction of a competing theory, in 1604. Kepler, whose primary interest was astronomy, undertook the study of optics as a way of better understanding and controlling errors introduced by human vision into astronomical measurements. This led to the idea, original with Kepler as far as I know, that we perceive distance from the convergence of the eyes in binocular vision, by a sort of natural geometry based on triangulation (see Figure 13):

Since to each animal a pair of eyes is given by nature, with a certain distance between them, by this support the sense of vision is most rightly used to judge the distances of Visibles, provided that that distance have a perceptible ratio to the distance of the eyes….

For, given two angles of a triangle, with the side between them, the remaining sides are given. (Kepler, 2000/1604, p. 79)

Kepler suggested that we could also get the equivalent information from one eye by moving it back and forth, that is, from absolute motion parallax. Finally, he suggested that a similar form of triangulation on incoming rays could be done with only one eye by using the width of the pupil as the base of the triangle; this form of potential information was transformed by the time of Descartes *Optics* in 1637 into the sensing of the accommodation of the eye. Descartes also added blur, combined with image intensity, as a means of perceiving relative depth (Descartes, 1637/1965, pp. 105–107).

Kepler, the astronomer, and Descartes, the creator of analytic geometry, thus laid out the theory of cues to distance perception through an abstract empty space. With a few additions, such as the demonstration of stereopsis by Wheatstone in 1838 and the description of relative motion parallax by Helmholtz, it was the theory of Kepler and Descartes that was inherited and rejected by Gibson.

Did al-Haytham's ground theory vanish without leaving any trace behind? The short title of Kepler's 1604 book is *Additions to Witelo*. It is primarily based on a careful study and critique of a 1572 edition of Witelo's optics that included the Latin translation of al-Haytham's optics, and Kepler frequently mentions al-Haytham as well as Witelo. Thus, it seems almost certain that Kepler was well acquainted with al-Haytham's ground theory. As far as I have found, however, he makes no mention of it, nor does Descartes, who also seems likely to have been acquainted with it.^11^ Nor, following Descartes, do I know of any explicit mention of al-Haytham's ground theory until John White's discussion of it in connection with the Renaissance depiction of pictorial space (White, 1957).

Nevertheless, in the early 17th century, there was such widespread activity studying and developing optical systems, which involved the interplay of telescopes and human vision, that it seems unlikely that the transition from al-Haytham's ground theory to the more abstract, mathematical theory of Kepler and Descartes could have happened all at once. A finer-grained history of that period may well reveal a more complex transition.^12^

Some fragments of al-Haytham's theory may have remained in circulation. For example, Berkeley writes about learning to associate the distance of an object with its visual appearance through the experience of walking up to it (Berkeley, 1709/1910).^13^ Some theories in the endless debates about the moon illusion may have been borrowed or adapted from al-Haytham (Ross & Plug, 2002). Finally, lists or brief discussions of the monocular cues to distance have often included an object's height in the visual field; this cue obtains whatever validity it has from the constraint that the object is resting on the ground (or its equivalent, such as a floor).^14^ Even this cue of height in the field, however, was relegated to a secondary role in distance perception—one of the pictorial cues—as distinct from the primary roles assigned to binocular vision and motion. Al-Haytham's ground theory, if not entirely lost, was sufficiently fragmented and dispersed to make Gibson's rediscovery of it, some 900 years after it was first postulated, seem like a “radical reformulation” (Gibson, 1950, p. 6) of existing theories to him and to many others who have been influenced by his work.

To sum up, Ibn al-Haytham's “ground theory” (c. 1039 AD) offers a detailed, rigorous, empirically oriented explanation of distance perception. It may be the first essentially modern, scientific theory of distance perception. It was the dominant theory of distance perception in Europe for about 400 years (c. 1220–1604). It was abandoned and largely forgotten, as a coherent and comprehensive theory, after about 1604. And finally, a remarkably similar theory was independently described by Gibson in 1950.

## Notes

1. An earlier version of this paper was presented in a symposium on al-Haytham's contributions to visual perception at the 2001 European Conference on Visual Perception in Kusadasi, Turkey.

2. Al-Haytham's ground theory is here described as a theory of *distance* perception rather than a theory of *space* perception, as Gibson described his theory. This is in part because my discussion here is limited to distance perception, but more importantly, it would be an anachronism to imply that the philosophical/scientific concept of *space* existed in al-Haytham's time as it did in Gibson's. Recent works in the history of mathematics and of philosophy suggest that the philosophical/scientific concept of space did not take shape until the 17th and 18th centuries, beginning with the work of Kepler, Descartes, and others, coming to prominence in a dispute between Newton and Liebnitz concerning the nature of space, and maturing in Kant's theory of the *a priori* intuition of space (De Risi, 2015; Janiak, 2020). The more specific concept of *visual space perception* appears to have arisen in response to these broader developments and the zeitgeist they engendered, but a survey of this history is far beyond the scope of the present paper. Gibson clearly intended to substitute his ground theory for the existing concept of visual space perception. He wrote “The conception of an empty space of three dimensions was a conception of philosophers and physicists. It was appropriate for an analysis of the abstract world of events defined by Newton. It was and still is of enormous value for analysis in the physical sciences. But the fact that it simplifies such problems does not make it the best starting point for the problem of visual perception.” (Gibson, 1950, p. 60).

3. All page number citations in this section are to Gibson (1950).

4. By “the classical theories of space perception,” Gibson evidently means the predominant theories from the 18th century up to the time of his writing.

5. All page number citations in this section are to al-Haytham (1989).

6. Why al-Haytham specifies a “narrow hole” here is an interesting question. Clearly, his principal intention, in limiting vision here, is to prevent the observer from seeing the ground on the other side of the hole in the wall, and so to prevent that ground surface from being used to perceive the distances, and separation in depth, of the two walls on the other side of the hole. It is also possible, however, that the “narrow hole” is also intended to prevent binocular disparity and/or motion parallax, either of which might enable some perception of depth separation even when the ground was not visible. Al-Haytham's restriction of vision here would then be like the peephole through which one views the Ames Room or other perceptual displays that only work with monocular, stationary vision.

7. Wu et al. (2004) have presented research that they interpret as showing that the process of perceiving distance over the ground involves the sequential integration of local patches of ground, beginning near the observer and proceeding into the distance. This hypothesized process clearly bears some resemblance to the process that al-Haytham is describing. There is also, however, an important difference in that the hypothesis of Wu et al. concerns the perceptual process that occurs *every time* one perceives distance over the ground, but al-Haytham is discussing the initial process of *learning* to calibrate perceived distance, beginning with looking down at one's feet and gradually extending, through experience, to larger and larger distances; he does not suggest that this process would be repeated every time that distance over the ground is perceived.

8. As can be seen in these translated quotes from Euclid, what is seen is described using the word “appear.” In the literature of space perception, the word “appear” is ambiguous (Sedgwick, 1986, p. 21–8). “Appear” can refer either to the perceived visual world or to the perceived visual field, to use Gibson’s (1950) distinction. Observers can attend to either, with more or less success depending on the context. Although Euclid's “appear” is sometimes read as referring to the perceived visual world, to do so involves Euclid in such gross errors of perception as to make that reading unlikely. It is more plausible that Euclid's “appear” is referring to the perceived visual field.

9. Bacon's reliance on al-Haytham seems clear in this example of the two walls. This impression is verified in the following paragraph of Bacon's text—a discussion of the perceived height of clouds—which quotes directly from “Alhacen” (the Medieval Latin version of al-Haytham's name), thus making Bacon's use of al-Haytham's text explicit.

10. I thank Prof. Mary Baine Campbell for her translation from the Latin of the quoted passage from Witelo.

11. Why neither Kepler nor Descartes mention al-Haytham's ground theory is a key question in the development of the concept of “space perception,” but to pursue it here is beyond the scope of this paper. Both Kepler, with his mathematically derived laws of planetary motion, and Descartes, with his analytical geometry, contributed substantially to what Koyré describes as the transition “from the closed world to the infinite universe” that accompanied the development and acceptance of the Copernican theory of the solar system (Koyré, 1957). Tentatively, I speculate that to Kepler, Descartes, and those who followed them, the ground theory of al-Haytham may have seemed ill-suited to the dawning concept of an abstract, mathematical, empty, and infinite space.

12. One important transitional figure is Aguilonius (1613). Working in the period between Kepler and Descartes, he may have been attempting a novel system that is in part a synthesis of the old and new theories. Best known for his work on the horopter (Howard, 2012, pp. 68–73), he also offers an extended discussion of distance perception. He argues for the importance of binocular vision for near tasks done in empty air but also acknowledges (implicitly following al-Haytham) the role of the continuous ground surface in perceiving larger distances. He proposes that the binocular process is used for near (“minor”) distances and the continuous ground process is used for far (“major”) distances (Aguilonius, 1613, p. 157, Proposition 5). (I thank Prof. Mary Baine Campbell for help with translations from the Latin of Aguilonius.)

13. Al-Haytham's use of the experiences of walking and reaching to calibrate distance perception is resembled by Bishop Berkeley's much later “new theory” of vision (Berkeley, 1709/1910), but there is a crucial difference in that Berkeley's theory was solidly in the camp of those later theories that Gibson refers to as “classical.” Berkeley wrote “It is, I think, agreed by all that distance, of itself and immediately, cannot be seen. For distance being a Line directed end-wise to the eye, it projects only one point in the fund of the eye, which point remains invariably the same, whether the distance be longer or shorter” (Berkeley, 1910/1709, p. 1).

14. For an extended discussion of this point, see Sedgwick (2021, pp. 25–26).
